# Geometrical Optimization and Transverse Thermoelectric Performances of Fe/Bi_2_Te_2.7_Se_0.3_ Artificially Tilted Multilayer Thermoelectric Devices

**DOI:** 10.3390/mi13020233

**Published:** 2022-01-30

**Authors:** Hongyu Zhou, Huang Liu, Guoping Qian, Huanan Yu, Xiangbing Gong, Xi Li, Jianlong Zheng

**Affiliations:** National Engineering Laboratory for Highway Maintenance Technology, School of Traffic and Transportation Engineering, Changsha University of Science and Technology, Changsha 410114, China; lhcsust@163.com (H.L.); huanan.yu@csust.edu.cn (H.Y.); xbgong@csust.edu.cn (X.G.); csu_lixi@126.com (X.L.); zjl@csust.edu.cn (J.Z.)

**Keywords:** Fe/Bi_2_Te_2.7_Se_0.3_, rational design, transverse thermoelectric performances

## Abstract

Transverse thermoelectric performance of the artificially tilted multilayer thermoelectric device (ATMTD) is very difficult to be optimized, due to the large degree freedom in device design. Herein, an ATMTD with Fe and Bi_2_Te_2.7_Se_0.3_ (BTS) materials was proposed and fabricated. Through high-throughput calculation of Fe/BTS ATMTD, a maximum of calculated transverse thermoelectric figure of merit of 0.15 was obtained at a thickness ratio of 0.49 and a tilted angle of 14°. For fabricated ATMTD, the whole Fe/BTS interface is closely connected with a slight interfacial reaction. The optimizing Fe/BTS ATMTD with 12 mm in length, 6 mm in width and 4 mm in height has a maximum output power of 3.87 mW under a temperature difference of 39.6 K. Moreover the related power density per heat-transfer area reaches 53.75 W·m^−2^. This work demonstrates the performance of Fe/BTS ATMTD, allowing a better understanding of the potential in micro-scaled devices.

## 1. Introduction

Thermoelectric (TE) technology is well-known for its capability to directly convert heat into electricity, and it has a great value in power generation, cooling, thermal detection, etc. [1,2,3]. The performance of TE technology is determined by the figure of merit (ZT) [4]. Over the past two decades, great advancements, including band-structure engineering [5,6,7], phonon engineering [8,9,10,11] and magnetoelectric engineering [12,13], have been proposed to enhance the ZT values of traditional TE materials. Nevertheless, the parallel or anti-parallel relationship between the electrical current (I) and the heat flow (Q) impedes the progress of optimizing the transport parameters in an individual way to the higher ZT values. Beyond that, the traditional TE devices perform complex π-type structure. Multiple n-type TE legs and p-type TE legs are connected electrically in series through metal electrodes to enlarge the TE electromotive force, thus hindering their miniaturization to meet the requirements of microelectronic applications [14,15].

Transverse TE counterparts have been proposed as an alternative approach, whereby it can generate off-diagonal element I and Q [16,17]. The I and Q are perpendicular to one another; the electrical conductivity, thermal conductivity, and Seebeck coefficient are anisotropic. It brings three distinct advantages. First, there is a greater degree of freedom in optimizing the transport parameters in an individual way. Second, the related transverse TE device is single-leg, and no electrode required. It can be cut into a variety of shapes, such as tubes, sheets, thin film, trapezoid, and cone, to fit the demand of infinite-stage cascade power generator or cooler [18,19,20]. Third, the dependence of V_x_ on the length to height ratio is greatly beneficial in developing the micro/nano-scaled devices. It works best for the extremely long and thin device [21,22,23,24].

One of the transverse TE counterparts, the artificially tilted multilayer thermoelectric device (ATMTD), has gained increasing attention, because, in addition to the three distinct advantages above, the artificial combination can be almost any material [25,26]. This gives more options to explore the high-performance ATMTD. To establish the off-diagonal element, both combinatorial materials in the ATMTD must be stacked together in an alternating pattern and then turned at a tilted angle (θ) to be effective in the anisotropic Seebeck coefficient. The thickness ratio (δ) of each combinatorial material also makes its own contribution to the anisotropy of electrical and thermal conductivity. The concerned performance of the transverse figure of merit (ZT_zx_) in ATMTD is defined as ZT_zx_ = σ_xx_S_zx_^2^T/κ_zz_, where the ZT_zx_, σ_xx_, S_zx_, κ_zz_ and T are the transverse figure of merit, transverse electrical conductivity, transverse Seebeck coefficient, transverse thermal conductivity and absolute temperature, respectively [27]. Recently, many efforts have been made to combine different materials to study the ZT_zx_ of ATMTD, such as Al/Si [28], Bi/Cu [29], Pb/Bi_2_Te_3_ [30], Ni/Bi_0.5_Sb_1.5_Te_3_ [31,32,33], YbAl_3_/Bi_0.5_Sb_1.5_Te_3_ [34] and Bi/Bi_0.5_Sb_1.5_Te_3_ [24]. However, the geometrical configurations (θ and δ) of the ATMTD also play a very important role in improvement of ZT_zx_, since it takes advantage of the transverse TE effect in an artificially tilted multilayer structure. A rational design for the given combinations that guides the ZT_zx_ optimization is highly desired.

In this study, n-type Bi_2_Te_2.7_Se_0.3_ (BTS) and p-type pure irons (Fe) were selected as the combinatorial materials for a new ATMTD. Because BTS has a large Seebeck coefficient, small electrical and thermal conductivities near room temperature, Fe has a small Seebeck coefficient, large electrical and thermal conductivities [35]. The large different values for TE parameters in BTS and Fe are beneficial to its ZT_zx_ [36]. To exploit the best geometrical configurations of the given Fe/BTS ATMTD, a high-throughput calculation was performed. A Fe/BTS ATMTD of 12 mm in length, 6 mm in width and 4 mm in height was fabricated with the optimized tilted angle of 14° and optimized thickness ratio of 0.49. The results reveal that the experimental S_zx_ value is in good agreement with theoretical ones. The maximum output power and related power density per heat-transfer area are about 3.87 mW and 53.75 Wm^−2^ under a temperature difference of 39.6 K, respectively.

## 2. Methods of Calculation and Experiment

### 2.1. Theoretical Calculation Model of Fe/BTS ATMTD

**Figure 1:**
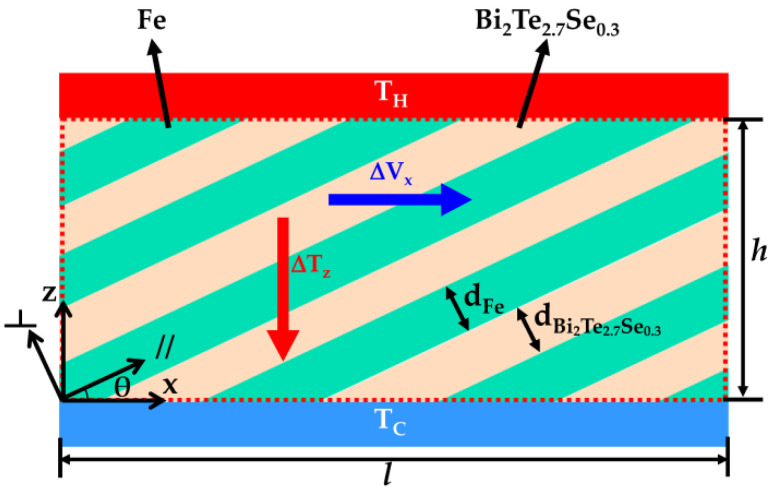
Simplified model of the Fe/BTS ATMTD from the sectional view.

The structure of ATMTD contains two alternately stacked layers, Fe and BTS, with thickness d_Fe_ and d_BTS_. After the stacking layer plane is tilted at an angle, θ, against the ATMTD surface (θ ≠ 0° or 90°), the anisotropy of transport parameters comes from two alternately stacked layers, parallel and perpendicular stacking-layer planes, as well as tilt. This means that the Seebeck coefficient of Fe/BTS ATMTD is anisotropic and the off-diagonal Seebeck coefficient of Fe/BTS ATMTD is non-zero, which provides the theoretical background to see transverse TE effect. As shown in Figure 1, when a temperature gradient (ΔT_z_) in Fe/BTS ATMTD is established along the *z*-axis under the hot-side temperature (T_H_) and cold-side temperature (T_C_), it will yield a transverse TE electromotive force (ΔV_x_) along the *x*-axis. The ΔV_x_ can be expressed as follows:

ΔV_x_ = S_zx_ΔT_z_l/h (1)

where l and h are the length and the height of Fe/BTS ATMTD, respectively; and S_zx_ is the difference of Seebeck coefficient in the *z*-axis and *x*-axis direction of Fe/BTS ATMTD.

The ΔV_x_ and other TE performance of Fe/BTS ATMTD are relevant to ZT_zx_ = σ_xx_S_zx_^2^T/κ_zz_. To maximum ZT_zx_ values, we begin with a description of the off-diagonal elements (ρ_xx_, κ_zz_, and S_zx_) along the different directions. It can be described by the tensors (M):

(2) $$\left[M\right]=\left[\begin{array}{ccc} M_{//}\cos^{2}{\theta +M_{⟂}}_{}\sin^{2}\theta & 0 & \left(M_{//}-{\;M_{⟂}}_{}\right)\sin\theta \cos\theta \\ 0 & M_{//} & 0\\ \left(M_{//}-M_{⟂}\right)\sin\theta \cos\theta & 0 & M_{//}\sin^{2}{\theta +M_{⟂}}_{}\cos^{2}\theta \end{array}\right]$$

Then, the σ_xx_, κ_zz_ and S_zx_ can be given as a function with tilted angle, θ, of the stacking layer plane in Fe/BTS ATMTD, which includes σ_//_, σ_⊥_, S_//_, S, κ_//_, κ_⊥_ and θ.

σ_xx_ = 1/ρ_xx_ = 1/(ρ_//_cos^2^θ + ρ_⊥_sin^2^θ) = σ_//_σ_⊥_/(σ_⊥_cos^2^θ + σ_//_sin^2^θ) (3)

S_zx_ = (S_//_ − S_⊥_)sinθcosθ (4)

κ_zz_ = κ_//_sin^2^θ + κ_⊥_cos^2^θ (5)

To illustrate how transport behaviors arise in alternately stacked layers, we considered the simplified model below. Assume that the electrical and thermal contact resistances in the Fe/BTS ATMTD could be ignored. Combined with Kirchhoff’s theory [28], the electrical conductivity (σ_//_, σ_⊥_), Seebeck coefficient (S_//_, S_⊥_) and thermal conductivity (κ_//_, κ_⊥_) parallel and perpendicular to the stacking layer plane become as follows:

σ_//_ = δσ_Fe_ + (1 − δ)σ_BTS_, σ_⊥_ = σ_Fe_σ_BTS_/[(1 − δ)σ_Fe_ + δσ_BTS_] (6)

S_//_ = [δS_Fe_σ_Fe_ + (1 − δ)S_BTS_σ _BTS_]/[δσ_Fe_ + (1 − δ)σ_BTS_] (7)

S_⊥_ = [δS_Fe_κ_BTS_) + (1 − δ)S_BTS_κ_Fe_]/[(1 − δ)κ_Fe_ + δκ_BTS_) (8)

κ_//_ = δκ_Fe_ + (1 − δ)κ_BTS_, κ_⊥_ = κ_Fe_κ_BTS_/[(1 − δ)κ_Fe_ + δκ_BTS_] (9)

where the subscripts “//” and ‘‘_⊥_” denote the directions parallel and perpendicular to the stacking layer plane of the Fe/BTS ATMTD. Moreover, σ_Fe_, σ_BTS_, S_Fe_, S_BTS_, κ_Fe_ and κ_BTS_ are the electrical conductivity of Fe, electrical conductivity of BTS, Seebeck coefficient of Fe, Seebeck coefficient of BTS, thermal conductivity of Fe and thermal conductivity of BTS, respectively; and δ = d_Fe_/(d_Fe_ + d_BTS_) represents the thickness ratio of Fe layer in Fe/BTS ATMTD.

Note that both the TE parameter (σ_Fe_, σ_BTS_, S_Fe_, S_BTS_, κ_Fe_ and κ_BTS_) of constituent materials and the geometrical configuration (δ and θ) of Fe/BTS ATMTD are the key factor to maximum ZT_zx_ values. The solution for ZT_zx_ is complex. A high-throughput calculation to investigate the relevance between the transverse TE properties is very important.

### 2.2. Experimental Procedure of Fabricated Fe/BTS ATMTD

**Figure 2:**
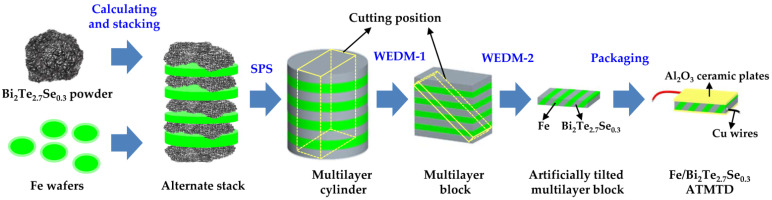
Sketch map of the processes of fabricated Fe/BTS ATMTD.

The Fe/BTS ATMTD was fabricated by using spark plasma sintering (SPS) and two-step wire-cut electrical discharge machining (WEDM) method. The specific processes are presented in Figure 2. Firstly, it was necessary to optimize the geometrical configuration (δ and θ) of Fe/BTS ATMTD with theoretical calculation before its manufacture to maximize ZT_zx_ value. Secondly, the starting materials, including the Fe wafers and BTS powders, were alternately loaded in graphite die. The thicknesses of layers Fe and BTS were identical to the designed δ value. Thirdly, the alternately stacked material was sintered into a multilayer cylinder by SPS at 673 K for 10 min, under a pressure of 40 MPa. Fourthly, the multilayer cylinder was shaped into an artificially tilted multilayer block, and the cutting position was controlled by the two-step WEDM method to realize the desired θ value. Finally, the Fe/BTS ATMTD with the optimized δ and θ was obtained via soldering Cu wires and bonding Al_2_O_3_ ceramic plates on the artificially tilted multilayer block.

### 2.3. Characterization and Performance Evaluation of Fe/BTS ATMTD

The microstructure and element distribution at the interface were characterized by electron probe microanalysis (EPMA, JEOL JXA-8230) equipped with an X-ray spectroscopy detector. The power generation performance of Fe/BTS ATMTD was evaluated at a temperature difference from 10 to 40 K, using a self-made measuring equipment that was presented in our previous report [33,34].

## 3. Results and Discussion

### 3.1. Geometrical Configuration Determination of Fe/BTS ATMTD

To quickly screen the maximum ZT_zx_ from a large number of combinations from δ and θ, a high-throughput calculation for σ_xx_, S_zx_, κ_zz_ and ZT_zx_ was implemented under different δ and θ values. The room-temperature electrical conductivity (σ), Seebeck coefficient (S), and thermal conductivity (κ) of Fe and BTS in the calculation model refer to Table 1.

**Figure 3:**
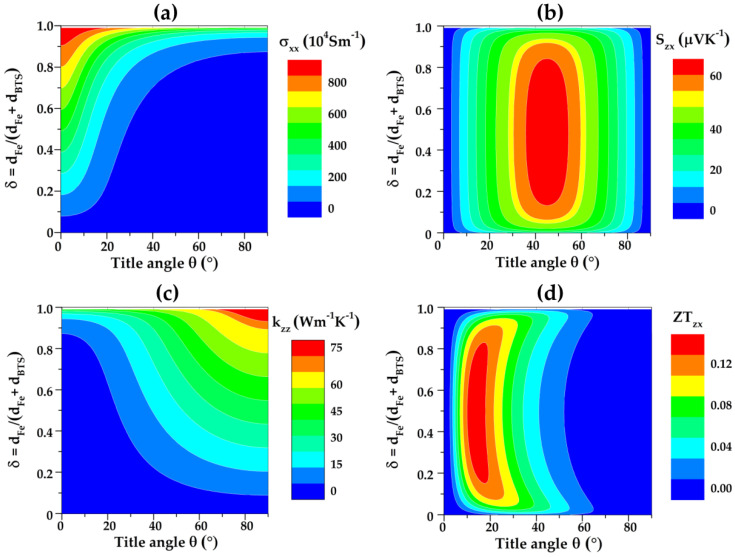
Thickness ratio (δ) and tilted angle (θ) dependences of (**a**) electrical conductivity σ_xx_; (**b**) transverse Seebeck coefficient S_zx_; (**c**) thermal conductivity κ_zz_; and (**d**) transverse figure of merit ZT_zx_ of Fe/BTS ATMTD.

Figure 3 shows the contour maps of Fe/BTS as a function of δ and θ. It can be seen that the σ_xx_ gradually increases with increasing the δ value from 0 to 1, due to the increased thickness of high conductivity Fe layer in Fe/BTS ATMTD (Figure 3a). Moreover, σ_xx_ first decreases slowly when θ value is less than 20° and then decreases dramatically in the range of 20–90°. This reduction indicates that the small θ can enhance the electron transport of Fe/BTS ATMTD in the *x*-axis direction. Figure 3b shows the δ and θ dependence on the S_zx_. The positive S_zx_ values indicate a p-type conduction behavior of Fe/BTS ATMTD. With increasing the δ, S_zx_ first increases and then decreases. When the δ value is close to 0.5, a larger S_zx_ value can be found. S_zx_ increases slowly when θ value is less than 30°, and then increases dramatically in the range of 30–45°. Nevertheless, when the θ value is larger than 45°, a drop of S_zx_ occurs. The maximum S_zx_ value of 66.3 μVK^−1^ is obtained at δ = 0.48 and θ = 45°. Figure 3c displays the δ and θ dependence of the κ_zz_. The κ_zz_ gradually increases with increasing the δ. In contrast to σ_xx_, the κ_zz_ gradually increases with increasing the θ, meaning that the small θ will be reduced the thermal transport of Fe/BTS ATMTD in the *z*-axis direction. Thus, a choice of small θ is confirmed to simultaneously optimize electrical and thermal transport properties in Fe/BTS ATMTD. Figure 3d shows the δ and θ dependence of the ZT_zx_. By increasing the δ and θ, the ZT_zx_ value first increases and then decreases. The maximum ZT_zx_ value for Fe/BTS ATMTD is 0.15 with δ = 0.49 and θ = 14°, which is increased by 400% as compared with that with δ = 0.48 and θ = 45°. Therefore, a properly geometrical configuration is favorable for maximizing the ZT_zx_ value in the ATMTD.

### 3.2. Microstructure Characterization of Fe/BTS ATMTD

**Figure 4:**
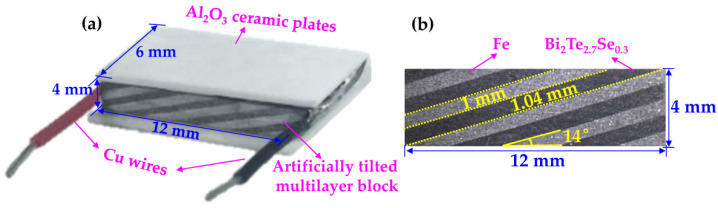
(**a**) Packaged Fe/BTS ATMTD; (**b**) cross-sectional image of artificially tilted multilayer block.

Based on the optimized δ and θ given by the high-throughput calculations, a packaged Fe/BTS ATMTD was fabricated (Figure 4a). The Fe/BTS ATMTD is rectangular, which is 12 mm in length, 6 mm in width and 4 mm in height. Figure 4b shows the cross-sectional image of artificially tilted multilayer block. The bright areas are Fe, while the black ones are BTS. The cross-sections with 10 alternately stacked layers of Fe and BTS are clearly visible. The whole Fe/BTS artificially tilted multilayer block is closely connected without macro-cracks. The thicknesses of Fe and BT layers are 1 mm and 1.04 mm, respectively, thus indicating that the δ value is 0.49. The as-prepared θ value is 14°. The as-prepared δ and θ values are consistent with the desired geometrical configuration.

**Figure 5:**
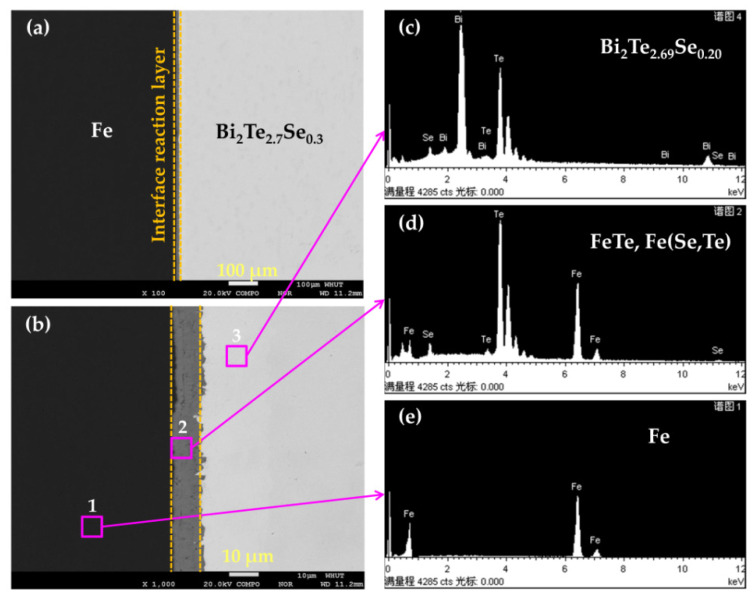
(**a**,**b**) BEI; (**c**,**d**,**e**) EDS results of Fe/BTS artificially tilted multilayer block.

Figure 5a presents a backscattered electron image (BEI) for the Fe/BTS artificially tilted multilayer block. It can be seen that the Fe/BTS artificially tilted multilayer block grows into a three-layer interfacial structure, which consists of Fe layer in the left side with a black color, interface reaction layer in the middle region with a dark gray color and BTS layer in the right side with a light gray color. The enlarged BEI images further manifest that there are three distinctive regions from the Fe layer to the BTS layer (Figure 5b). The Fe and BTS maintain excellent interface bonding, and no crack on the micrometer scale is observed. To figure out the composition of the three regions, we performed an energy-dispersive spectrometer (EDS) analysis (Figure 5c–e), and the corresponding average atomic ratios of Fe, Bi, Te and Se on the zones are listed in Table 2. The black area was confirmed to be Fe. The light gray area is Bi_2_Te_2.69_Se_0.20_, indicating a small amount of loss of Te and Se during the preparation of Fe/BTS ATMTD. The dark gray area in the middle region is speculated to be FeTe and Fe(Se, Te), which results from the interfacial reaction between Fe and BTS.

**Figure 6:**
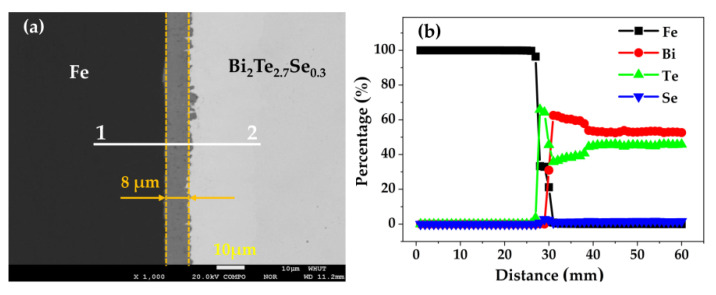
(**a**) BEI; (**b**) elemental line profiles of Fe, Bi, Te and Se elements at the Fe/BTS interface.

The BEI and line distributions of Fe, Bi, Te and Se elements were also conducted on the Fe/BTS interface by a wave-dispersive spectrometer (WDS), as shown in Figure 6. It is clear that three-layer interfacial structure is present from the Fe to BTS sides. The line distributions show that all the elements have a small amount of diffusion at the interface. However, the thickness of interface reaction layer is only 8 μm, which means that the interfacial reaction between Fe and BTS is slight, SPS and two-step WEDM method is suitable for fabricating a high-quality Fe/BTS ATMTD.

### 3.3. Power Generation Performance of Fe/BTS ATMTD

**Figure 7:**
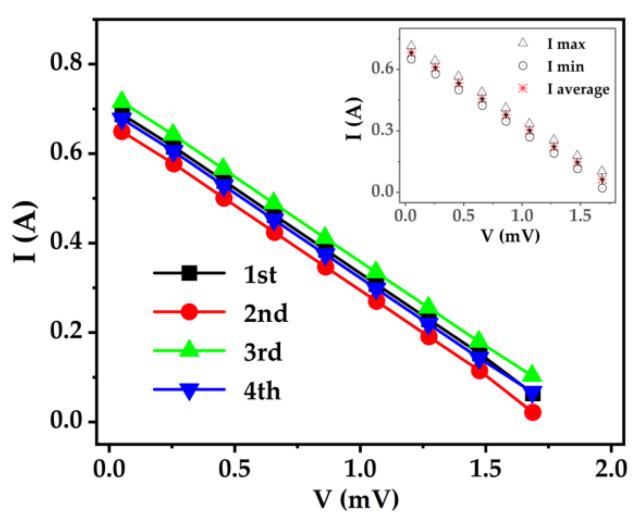
V–I curve of Fe/BTS ATMTTD at a fixed ΔT of 20 K after being testing four times. The inset shows the differences with the self-made measuring equipment.

Prior to recording the power-generation performance, a V–I measurement was performed for the packaged Fe/BTS ATMTD at a fixed ΔT of 20 K in the self-made measuring equipment (Figure 7). The inset shows the differences from reliability tests. The deviation within 5% indicates a good reproducible behavior in self-made measuring equipment.

**Figure 8:**
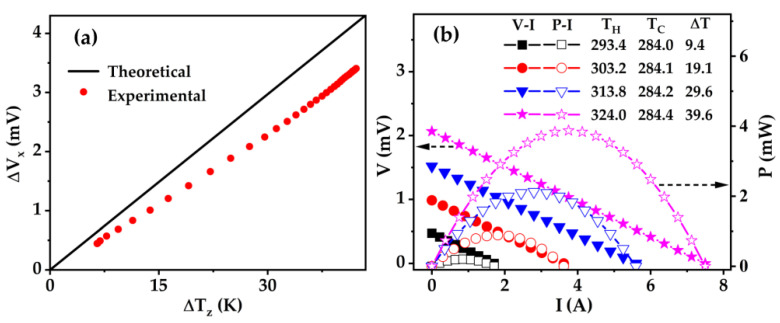
Power-generation performance of Fe/BTS ATMTTD: (**a**) ΔT_z_ dependence of ΔV_x_; (**b**) I dependences of V and P for Fe/BTS ATMTD.

To characterize the power generation performance of the as-prepared Fe/BTS ATMTD, the electrical output characteristics were evaluated with the self-made measurement system under various ΔT_z_ values. The theoretical ΔV_x_ values were estimated according to the equation ΔV_x_ = S_zx_ΔT_z_l/h, where S_zx_ is the transverse Seebeck coefficient of the as-prepared Fe/BTS ATMTD, l is the length of the as-prepared Fe/BTS ATMTD, h is the height of the as-prepared Fe/BTS ATMTD and ΔT_z_ is the temperature difference along the z direction. It can be seen that a ΔV_x_ along the x direction has generated when a ΔT_z_ applies along the z direction, clearly demonstrating the transverse Seebeck effect (Figure 8a). The experimental S_zx_ is 27 μV/K, which is within error to the theoretical ones of 30 μV/K [34]. The ΔV_x_ gradually increases with increasing the ΔT_z_. When ΔT_z_ = 9.4 K or 42.2 K, a maximum ΔV_x_ value is up to 0.7 mV and 3.4 mV, respectively. It may be noted that the ΔV_x_ value of over 7 V might be achieved in micro-scaled Fe/BTS ATMTD with h of 4 μm, l of 120 mm and ΔT_z_ of 9.4 K. The work voltage (V) and output power (P) as a function of work current (I) for Fe/BTS ATMTD under different ΔT are depicted in Figure 8b. The linear relationship of V and the parabolic variation of P as a function of I represents a typical feature of TE power generators, where the slope of V–I mean the internal resistance of Fe/BTS ATMTD. When the external resistance is matched with internal resistance, a peak P can be reached. The peak P of 0.20 mW is obtained under a TH of 293.4 K and ΔT of 9.4 K, and it increases with increasing ΔT. The maximum P is 3.87 mW for Fe/BTS ATMTD under TH of 324.0 K and ΔT of 39.6 K. The related power density per heat-transfer area is 53.75 W·m^−2^.

## 4. Conclusions

A high-throughput calculation was developed to rationally design the geometrical configuration of high-performance Fe/BTS ATMTD. It was revealed that the Fe/BTS ATMTD exhibits a maximum ZTz_x_ of 0.15 when δ = 0.49 and θ =14°. According to the optimized δ and θ, a Fe/BTS ATMTD of 12 mm in length, 6 mm in width and 4 mm in height was fabricated via the SPS and two-step WEDM method. Our microstructure analysis showed that the whole Fe/BTS interface is closely connected with a slight interfacial reaction. The thickness of interface reaction layer is only 8 μm. The maximum ΔV_x_ value is up to 0.7 mV and 3.4 mV when ΔT_z_ = 9.4 K and 42.2 K, respectively. The Fe/BTS ATMTD achieved a maximum P of up to 3.87 mW under a ΔT of 39.6 K, and the related power density per heat-transfer area reached 53.75 W·m^−2^. In the future, the performance of Fe/BTS ATMTD will be further improved if the height of the device is in the order of micron. This work demonstrates the versatile application of Fe/BTS ATMTD in power generation.

## Figures and Tables

**Table 1:** Room-temperature transport parameters of Fe and BTS.

Materials	σ (10^5^ S·m^−1^)	S (μ·V·K^−1^)	κ (W·m^−1^·K^−1^)
Fe	99 ^a^	15.0 ^b^	80.4 ^a^
BTS	1.4 ^a^	−122.1 ^a^	1.3 ^a^

^a^ Experimentally obtained by authors. ^b^ Reference [37].

**Table 2:** EDS results and speculated compositions of the different zones in Figure 5.

Zones	Atomic Ratio (%)				Speculated Composition
	Fe	Bi	Te	Se	
Fe	100	0	0	0	Fe
	53.04	0	43.25	3.71	FeTe, Fe(Se, Te)
BTS	0	40.95	55.01	4.04	Bi_2_Te_2.69_Se_0.20_
